# Comparative acute effects of mescaline, lysergic acid diethylamide, and psilocybin in a randomized, double-blind, placebo-controlled cross-over study in healthy participants

**DOI:** 10.1038/s41386-023-01607-2

**Published:** 2023-05-25

**Authors:** Laura Ley, Friederike Holze, Denis Arikci, Anna M. Becker, Isabelle Straumann, Aaron Klaiber, Fabio Coviello, Sophie Dierbach, Jan Thomann, Urs Duthaler, Dino Luethi, Nimmy Varghese, Anne Eckert, Matthias E. Liechti

**Affiliations:** 1 Clinical Pharmacology and Toxicology, Department of Biomedicine and Department of Clinical Research, University Hospital Basel, Basel, Switzerland; 2 Department of Pharmaceutical Sciences, University of Basel, Basel, Switzerland; 3 Psychiatric University Hospital, University of Basel, Basel, Switzerland; 4 Transfaculty Research Platform Molecular and Cognitive Neuroscience, University of Basel, Basel, Switzerland

**Keywords:** Psychology, Neurophysiology

## Abstract

Mescaline, lysergic acid diethylamide (LSD), and psilocybin are classic serotonergic psychedelics. A valid, direct comparison of the effects of these substances is lacking. The main goal of the present study was to investigate potential pharmacological, physiological and phenomenological differences at psychoactive-equivalent doses of mescaline, LSD, and psilocybin. The present study used a randomized, double-blind, placebo-controlled, cross-over design to compare the acute subjective effects, autonomic effects, and pharmacokinetics of typically used, moderate to high doses of mescaline (300 and 500 mg), LSD (100 µg), and psilocybin (20 mg) in 32 healthy participants. A mescaline dose of 300 mg was used in the first 16 participants and 500 mg was used in the subsequent 16 participants. Acute subjective effects of 500 mg mescaline, LSD, and psilocybin were comparable across various psychometric scales. Autonomic effects of 500 mg mescaline, LSD, and psilocybin were moderate, with psilocybin causing a higher increase in diastolic blood pressure compared with LSD, and LSD showing a trend toward an increase in heart rate compared with psilocybin. The tolerability of mescaline, LSD, and psilocybin was comparable, with mescaline at both doses inducing slightly more subacute adverse effects (12–24 h) than LSD and psilocybin. Clear distinctions were seen in the duration of action between the three substances. Mescaline had the longest effect duration (mean: 11.1 h), followed by LSD (mean: 8.2 h), and psilocybin (mean: 4.9 h). Plasma elimination half-lives of mescaline and LSD were similar (approximately 3.5 h). The longer effect duration of mescaline compared with LSD was due to the longer time to reach maximal plasma concentrations and related peak effects. Mescaline and LSD, but not psilocybin, enhanced circulating oxytocin. None of the substances altered plasma brain-derived neurotrophic factor concentrations. In conclusion, the present study found no evidence of qualitative differences in altered states of consciousness that were induced by equally strong doses of mescaline, LSD, and psilocybin. The results indicate that any differences in the pharmacological profiles of mescaline, LSD, and psilocybin do not translate into relevant differences in the subjective experience. ClinicalTrials.gov identifier: NCT04227756.

## Introduction

Psychedelic substances are capable of inducing exceptional alterations of consciousness. Mescaline, lysergic acid diethylamide (LSD), and psilocybin are some of the most prominent psychedelic representatives with similar purposes of use. Mescaline (the active component of Peyote and San Pedro cacti) and psilocybin (the active component of Psilocybe mushrooms) have been used for ethnomedical and spiritual rituals for centuries [1–3]. LSD and psilocybin are currently being investigated as therapeutic tools for the treatment of various psychiatric disorders [4–12]. All three substances are used recreationally around the world [13–16]. Mescaline, LSD, and psilocybin exert their mind-altering actions primarily via the stimulation of serotonin 5-hydroxytryptamine-2A (5-HT_2A_) receptors and are thus considered “classic hallucinogens”. However, they also exhibit differences in their pharmacological receptor profiles and pharmacokinetic properties. Mescaline binds to 5-HT_2A_, 5-HT_1A_, and adrenergic α_2A_ receptors in a similar concentration range. LSD most potently stimulates 5-HT_2A_ receptors and also 5-HT_2B/2C_, 5-HT_1A_, and dopamine D_1-3_ receptors. Psilocin (the active metabolite of psilocybin) stimulates 5-HT_2A_ receptors and simultaneously inhibits the serotonin transporter (SERT) [17]. Mescaline has been reported to be approximately 30 times less potent than psilocybin and 1000–3000 times less potent than LSD [17–19]. Mescaline is thus used at higher doses than LSD and psilocybin. Mescaline also reportedly has a delayed onset of action, possibly because of slow brain penetration [20]. The subjective effect duration (10–12 h) of a moderate mescaline dose (200–400 mg mescaline sulfate) has been reported to be similar to that of a moderate dose (0.1 mg) of LSD, clearly exceeding the duration of acute psilocybin effects (4–6 h) [18]. However, a direct blinded comparison of the substances and evaluations of pharmacodynamics and pharmacokinetics has not yet been validly determined in a modern clinical study. To date, it is unknown whether the partly distinct pharmacological profiles of the classic psychedelics mescaline, LSD and psilocybin actually translate into distinct subjective effects in humans. Early studies that compared serotonergic psychedelics were sparse and methodologically limited [21–23]. A study that directly compared the acute effects of LSD and psilocybin was recently published [24], but investigations of mescaline have been largely neglected in recent decades despite its widespread use in religious practices and therapeutic potential. Thus, the present study was a randomized, double-blind, placebo-controlled, cross-over trial in healthy participants that directly compared the acute effects of mescaline, LSD, and psilocybin. The main objective of the study was to detect potential pharmacological, physiological, and subjective/phenomenological differences between these three substances when used at doses that are equivalent in terms of the overall intensity of psychoactive effect, in order to facilitate interpretations across existing clinical trials and guide future designs and dose-finding for both research and therapeutic use. Furthermore, the involvement of oxytocin and brain-derived neurotrophic factor (BDNF) is frequently discussed in the context of potentially therapeutic mechanisms underlying the effects of psychedelics. BDNF increase following the administration of classic psychedelics has been shown to promote neuroplasticity [25]. Oxytocin release has been shown to facilitate social interaction, affiliation, and cognitive emotion regulation [26]. Both oxytocin and BDNF may thus contribute to therapeutic efficacy. 200, but not 100 µg LSD were reported to significantly increase BDNF plasma levels [27, 28]. Data on the oxytocin release through mescaline is currently lacking. In the present study, we hypothesized that the acute psychedelic effects induced by mescaline, LSD, and psilocybin would not be distinguishable using established psychometrics. We also predicted longer acute effects of mescaline > LSD > psilocybin due to pharmacokinetic differences.

## Methods

### Study design

This study used a double-blind, placebo-controlled, crossover design with four experimental test sessions to investigate responses to (i) 300 mg or 500 mg mescaline, (ii) 100 µg LSD, (iii) 20 mg psilocybin, and (iv) placebo. The order of administration was random and counterbalanced. Washout periods in between sessions were at least 10 days. The mescaline dose was increased from 300 mg in participants 1–16 to 500 mg in participants 17–32 after it became apparent in the first few study sessions that the initially chosen 300 mg dose was most likely lower than the LSD and psilocybin doses. Hence, allocation to the substance conditions (mescaline, LSD, psilocybin or placebo) was always random and blinded whereas, in contrast, allocation to the 300 or 500 mg mescaline dose was neither randomized nor blinded (i.e. participants 1–16 were informed that they would receive 300 mg mescaline and participants 17–32 were informed that they would receive 500 mg mescaline). The study was conducted in accordance with the Declaration of Helsinki and International Conference on Harmonization Guidelines in Good Clinical Practice, and was approved by the Ethics Committee of Northwest Switzerland (EKNZ) and the Swiss Federal Office for Public Health. The study was registered at ClinicalTrials.gov (NCT04227756).

### Participants

Thirty-two healthy participants (16 men and 16 women; mean age ± SD: 29 ± 4 years; range: 25–44 years) were recruited from a pool of volunteers who had contacted our research group with interest in participating in a trial that investigates psychedelics. All participants provided written informed consent and received payment for their participation. Exclusion criteria comprised age < 25 years or > 65 years, pregnancy and/or breastfeeding, personal or family (first-degree relative) history of major psychiatric disorders (assessed by the Semi-structured Clinical Interview for *Diagnostic and Statistical Manual of Mental Disorders*, 4th edition, Axis I disorders executed by a psychologist or physician), use of medication that may interfere with the study medication (e.g., antidepressants, antipsychotics, sedatives), chronic or acute physical illness (e.g., abnormal physical exam, electrocardiogram, or hematological and chemical blood analyses), excessive tobacco smoking (>10 cigarettes/day), lifetime prevalence of psychedelic drug use >10 times, illicit drug use within the last 2 months (except for Δ^9^-tetrahydrocannabinol), and illicit drug use during the study period. Participants were required to consume no more than 10 standard alcoholic beverages per week and to have no more than one drink on the day prior to the test sessions. Twenty participants (63%) had previously used a psychedelic, including mescaline (two participants, 1–3 times), LSD (12 participants, 1–4 times), psilocybin (12 participants, 1–5 times), ayahuasca (two participants, 2–5 times), 5-methoxy-*N,N*-dimethyltryptamine (5-MeO-DMT; one participant, twice), and 4-bromo-2,5-dimethoxyphenethylamine (2C-B; two participants, 1–2 times). Twenty-two participants (69%) had previously used 3,4-methylendioxymethamphetamine (MDMA; 1–30 times). 18 participants (56%) had used a stimulant, including amphetamine (13 participants, 1– approx. 50 times), cocaine (nine participants, 1– approx. 100 times), and methylphenidate (one participant, twice). Seven participants (22%) had used amyl nitrite (1–20 times), three (9%) had used ketamine (2–5 times). Three participants (9%) had used opiates (1–2 times). Five participants (16%) had never used any illicit drugs with the exception of cannabis.

### Study drugs

Mescaline hydrochloride (99.3% purity; ReseaChem GmbH, Burgdorf, Switzerland) was administered in opaque capsules that were produced according to Good Manufacturing Practice (GMP) in units that contained 100 mg mescaline. The exact analytically confirmed mescaline hydrochloride content (mean ± SD) was 95.0 ± 0.1 mg (*n* = 3 samples). The corresponding placebo consisted of identical opaque capsules that were filled with mannitol. LSD base (>99% purity; Lipomed AG, Arlesheim, Switzerland) was administered as an oral solution that was produced according to GMP in units that contained 100 µg LSD in 1 mL of 96% ethanol [29]. The exact analytically confirmed LSD base content was 92.5 ± 1.9 µg (*n* = 10 samples). The corresponding placebo consisted of identical vials that were filled with ethanol only.

Psilocybin (99.7% purity; ReseaChem GmbH, Burgdorf, Switzerland) was administered in opaque capsules that were produced according to GMP in units that contained 5 mg of psilocybin dihydrate. The exact analytically confirmed psilocybin content was 4.61 ± 0.09 mg (*n* = 10 samples). The corresponding placebo consisted of identical opaque capsules that were filled with mannitol.

The stability of all formulations was confirmed for the study duration. A double-dummy method was used. Participants 1–16 received seven capsules and one solution in each session: (i) seven placebo capsules and a placebo solution, (ii) four placebo capsules, three 100 mg mescaline capsules, and a placebo solution, (iii) seven placebo capsules and a 100 µg LSD solution, and (iv) three placebo capsules, four 5 mg psilocybin capsules, and a placebo solution. Participants 17–32 received nine capsules and one solution in each session: (i) nine placebo capsules and a placebo solution, (ii) four placebo capsules, five 100 mg mescaline capsules, and a placebo solution, (iii) nine placebo capsules and a 100 µg LSD solution, and (iv) five placebo capsules, four 5 mg psilocybin capsules, and a placebo solution. The participants were asked to guess which substance they had ingested during each study session at t = 3 h as well as after study completion.

### Study procedures

The study comprised a screening visit, five 25-h test sessions, and an end-of-study visit. The sessions were conducted in a calm hospital room. One participant and one or two investigators were present during each test session. The sessions began at 8:00 AM. A urine sample was taken to verify abstinence from drugs of abuse, and a urine pregnancy test was performed in women before each session. The participants then received a standardized breakfast (two croissants) and underwent baseline measurements. Mescaline, LSD, psilocybin, or placebo was administered at 9:00 AM. Outcome measures were repeatedly assessed for 24 h. From t = 2 h to t = 3 h, participants underwent a functional neuroimaging scan; its results are subject to a separate publication. The participants remained under constant supervision during the acute effect phase and an investigator spent the night in the room next to the participants. The participants were sent home the next day at approximately 9:15 AM.

### Subjective drug effects and effect durations

Subjective effects were assessed repeatedly using visual analog scales (VASs) [24, 27, 30, 31] 1 h before and 0, 0.25, 0.5, 0.75, 1, 1.5, 2, 3, 3.5, 4, 5, 6, 7, 8, 9, 10, 11, 12, 14, 16, and 24 h after drug administration. The Adjective Mood Rating Scale (AMRS) [32] was used 1 h before and 3, 6, 9, 12, and 24 h after drug administration. The 5 Dimensions of Altered States of Consciousness (5D-ASC) scale [33, 34] and the States of Consciousness Questionnaire (SOCQ) [35–37] were administered 24 h after drug administration to retrospectively rate psychedelic effects.

Time to onset, time to maximal effect, time to offset, and effect duration were assessed using individual effect-time plots of the VAS item “any drug effect” and an onset/offset threshold of 10% of the maximum individual response in Phoenix WinNonlin 8.3 (Certara, Princeton, NJ, USA) [29, 31].

### Autonomic and adverse effects

Blood pressure, heart rate, and tympanic body temperature were repeatedly measured at baseline and 0, 0.25, 0.5, 0.75, 1, 1.5, 2, 3, 3.5, 4, 5, 6, 7, 8, 9, 10, 11, 12, 14, 16, and 24 h after drug administration [38]. Pupil diameter was assessed at baseline and 1, 2, 4, 8, 12, and 24 h after drug administration [27]. Adverse effects were assessed 1 h before and 12 and 24 h after drug administration using the List of Complaints (LC) [39].

### Plasma mescaline, LSD, and psilocin concentrations

Blood was collected in lithium heparin tubes. Samples were centrifuged immediately and the plasma was then stored at −80 °C until analysis. Plasma mescaline [40], LSD [29], and psilocin [41] concentrations were determined by fully validated high-performance liquid chromatography tandem mass spectrometry.

### Circulating oxytocin and brain-derived neurotrophic factor

Plasma concentrations of oxytocin and brain-derived neurotrophic factor (BDNF) were assessed as described previously [27, 28, 30, 31]. Oxytocin levels were measured at baseline and 1.5, 3, and 6 h after drug administration. Plasma BDNF levels were measured at baseline and 3, 6, and 12 h after drug administration.

### Pharmacokinetic analyses

Pharmacokinetic parameters were estimated using non-compartmental methods as described previously [29]. The analyses were conducted using Phoenix WinNonlin 8.3 (Certara, Princeton, NJ, USA).

### Data analysis

Peak maximum effect (E_max_) and/or minimum effect (E_min_) or peak change from baseline (ΔE_max_) values were determined for repeated measures. The values were then analyzed using repeated-measures analysis of variance (ANOVA) with drug as the within-subjects factor, followed by Tukey post hoc tests using R 4.2.1 software (RStudio, PBC, Boston, MA, USA). The criterion for statistical significance was *p* < 0.05.

## Results

### Subjective drug effects

**Fig. 1:**
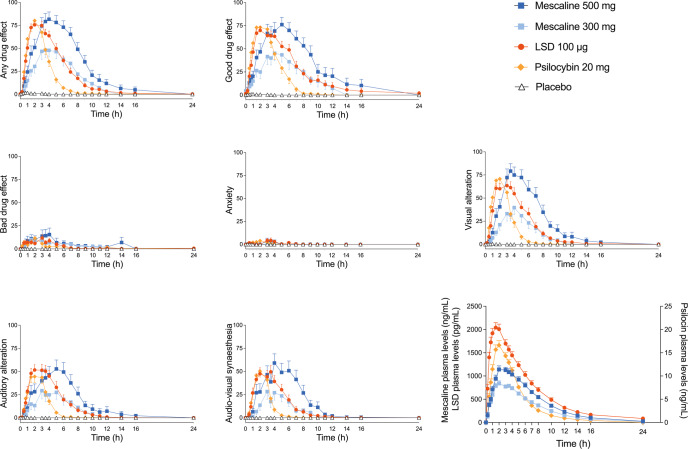
Acute subjective effects on the Visual Analog Scale (VAS) and plasma concentrations over time that were induced by mescaline (300 and 500 mg), LSD, psilocybin, and placebo. The 500 mg mescaline dose, LSD, and psilocybin induced similar subjective peak effects on all items. The low 300 mg mescaline dose induced lower peak effects than the high 500 mg mescaline dose, LSD, and psilocybin. The substances differed in their durations of action. Mescaline showed the longest effect duration of action compared with the other substances, followed by LSD and lastly psilocybin. The onset rates of subjective effects of LSD and psilocybin were comparable, whereas mescaline showed a slower onset and delayed peak of subjective effects. The substances were administered at t = 0 h. The data are expressed as the mean ± SEM ratings in 32 participants for LSD and psilocybin and in 16 participants for each mescaline dose. The corresponding statistics are presented in Supplementary Table S1.

**Fig. 2:**
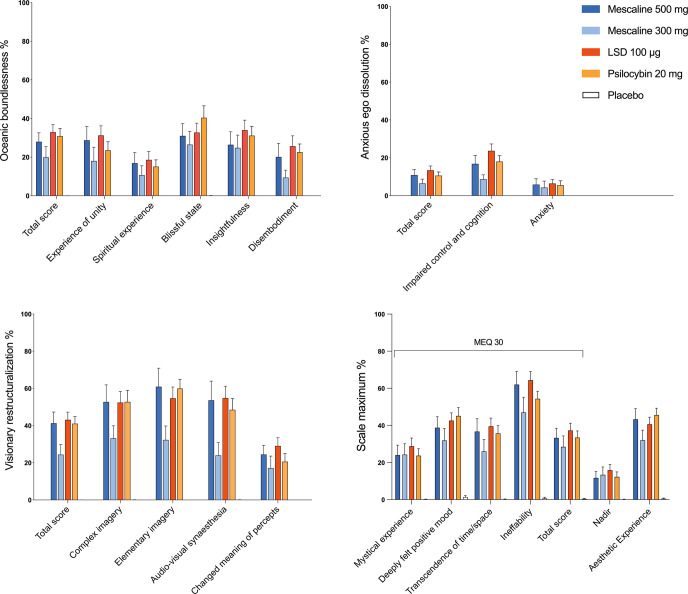
Acute alterations of mind, measured by the Five Dimensions of Altered States of Consciousness (5D-ASC) and the Mystical Experience Questionnaire (MEQ). The high 500 mg mescaline dose, LSD, and psilocybin induced comparable subjective effects on all subscales. The low 300 mg mescaline dose induced lower effects than all other drug conditions. Placebo scores did not reach the visualization threshold. The data are expressed as the mean ± SEM percentage of maximum scale scores in 32 participants for LSD and psilocybin and in 16 participants for each mescaline dose. The corresponding statistics are presented in Supplementary Tables S2 and S3.

Subjective effects over time assessed by the VASs are shown in Fig. 1 and Supplementary Fig. S1. Alterations of mind and mystical-type effects assessed by the 5D-ASC and MEQ are shown in Fig. 2. Effects on mood assessed by the AMRS are shown in Supplementary Fig. S2. The corresponding statistics are presented in Supplementary Tables S1–4. Overall, LSD, psilocybin, and the high 500 mg mescaline dose generated comparable subjective effects. There were no significant differences in maximum subjective effects ratings on the VAS, the 5D-ASC, the MEQ, or the AMRS between these three conditions. For the 300 mg mescaline dose, weaker effects were found on all four psychometric questionnaires compared with the high 500 mg mescaline dose, LSD, and psilocybin. Only on the AMRS, mescaline induced more ‘inactivity’ compared with psilocybin and placebo.

Descriptive parameters for the acute subjective response curves (VAS “any drug effect” over time) for each substance are shown in Table 1. Acute subjective effects of mescaline lasted longer than those of LSD (*n* = 32, *p* < 0.05) and psilocybin (*n* = 32, *p* < 0.001), and subjective effects of LSD lasted longer than those of psilocybin (*n* = 32, *p* < 0.001) (Table 1). The longer duration of action of mescaline compared with LSD was attributable to its slower onset (*n* = 32, *p* < 0.001), its longer time to maximal effect (*n* = 32, *p* < 0.001), and broader peak plateau of the subjective effect-time curve, whereas the comedown was equally fast (Table 1; Fig. 1). In addition to its delayed onset and later peak effect, the onset and t_max_ of the 500 mg mescaline effect showed larger interindividual variance compared with LSD and psilocybin (Table 1, Fig. 1).

**Table 1:** Characteristics of the subjective response to Mescaline, LSD, and Psilocybin.

Effect	Mescaline 300 mg	Mescaline 500 mg	LSD 100 µg	Psilocybin 20 mg
	*n* = 16	*n* = 16	*n* = 32	*n* = 32
Time to onset (h)	0.8 ± 0.5^+***##^ (0.4–1.9)	0.9 ± 0.6^+#^ (0.1–2.7)	0.4 ± 0.2 (0.0–1.0)	0.5 ± 0.3 (0.1–1.3)
Time to offset (h)	10.5 ± 1.9^+###^ (7.4–14)	12.0 ± 3.4^+**###^ (7.9–22)	8.6 ± 3.0^###^ (4.9.–19)	5.3 ± 1.7 (3.0–11)
Time to maximal effect (h)	4.0 ± 1.3^+***###^ (3.1–8.0)	3.4 ± 1.2^+*##^ (1.4–6.0)	2.3 ± 1.0 (0.75–4.0)	2.1 ± 1.0 (0.5–4.0)
Effect duration (h)	9.7 ± 2.2^+##^ (5.6–13)	11.1 ± 3.8^+*###^ (6.0–22)	8.2 ± 3.1^###^ (4.3.–19)	4.9 ± 1.7 (2.6–10)
Maximal effect (%)	58 ± 31*^#^ (0–100)	86 ± 27 (6–100)	83 ± 21 (29–100)	87 ± 17 (43–100)
AUEC	319 ± 223 (0–671)	616 ± 339^###^ (2.8–1507)	423 ± 205^#^ (64–863)	267 ± 91 (91–472)

Parameters are for “any drug effect” as determined using the individual effect-time curves. The threshold to determine times to onset and offset was set to 10% of the individual maximal response. Values are mean ± SD (range). *P < 0.05, **P < 0.01, ***P < 0.001 compared with LSD; ^#^*P* < 0.05, ^##^*P* < 0.01, ^###^*P* < 0.001 compared with psilocybin; Tukey tests; +*n* = 15; AUEC, area under the effect curve.

### Autonomic and adverse effects

**Fig. 3:**
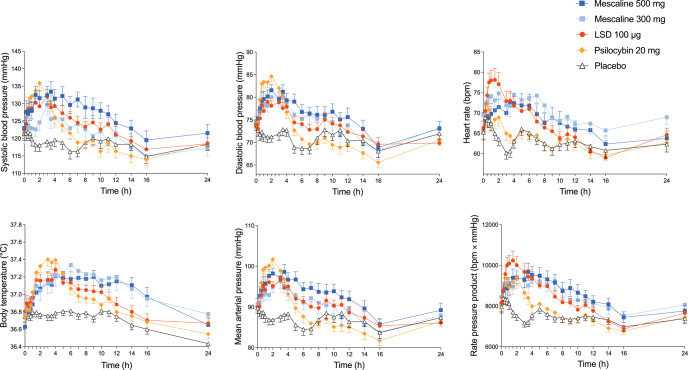
Acute autonomic effects. The high 500 mg mescaline dose, LSD, and psilocybin similarly increased systolic blood pressure, heart rate, body temperature, and the rate pressure product. LSD showed a significantly lower maximal diastolic blood pressure response compared with psilocybin. Conversely, LSD showed a trend toward an increase in heart rate compared with psilocybin. The data are expressed as the mean ± SEM of maximum responses in 32 participants for LSD and psilocybin and in 16 participants for each mescaline dose. The corresponding statistics are shown in Supplementary Table S5.

Autonomic effects over time and corresponding statistics are shown in Fig. 3 and Supplementary Table S5. Frequently reported adverse effects, as assessed by the List of Complaints and corresponding statistics are presented in Supplementary Tables S5–6. All three substances moderately increased systolic and diastolic blood pressure, body temperature, and pupil size relative to placebo. Among the three substances, only one significant difference was seen, i.e. psilocybin showed a higher diastolic blood pressure response compared with LSD. LSD showed a trend towards increases in heart rate and the rate pressure product compared with the other drug conditions. Autonomic effects coincided with the substances’ individual duration of action.

Mescaline (at both doses), LSD, and psilocybin similarly increased pupil size compared with placebo (Supplementary Fig. S3, Supplementary Table S5).

All drug conditions generated a higher total acute (0–12 h) adverse effect score on the List of Complaints compared with placebo (Supplementary Table S5). Only mescaline (*n* = 32) showed significant subacute (12–24 h) adverse effects relative to placebo. Adverse events during the study included severe headaches (three participants after mescaline, one participant after LSD, and one participant after psilocybin), fatigue (two participants after mescaline), ear congestion (one participant after LSD), nosebleed (one participant after mescaline), muscle twitches (one participant after psilocybin), and depressive symptomatology that lasted for several days to weeks (one participant after psilocybin and one participant after all three substances), which resolved spontaneously. A total of four flashback phenomena occurred (one participant after mescaline, [twice between the second and fourth week after the last study session] and one participant after LSD [twice within the first week following the second study session]). No serious adverse events occurred.

### Circulating oxytocin and BDNF

Effects on plasma levels of oxytocin and BDNF are shown in Supplementary Fig. S4 and Supplementary Table S5. Mescaline and LSD significantly increased plasma oxytocin levels compared with placebo. Oxytocin levels were significantly higher after mescaline compared with psilocybin. None of the substances altered plasma BDNF.

### Plasma drug concentrations

The concentration-time curves for mescaline, LSD, and psilocin and their metabolites are shown in Fig. 1 and Figure S5. Descriptive parameters of the acute subjective response and pharmacokinetic parameters of mescaline, LSD, and psilocybin are shown in Table 1 and Table 2, respectively. The geometric mean maximum (C_max_) values (range) for 300 and 500 mg mescaline were 858 (600–1284) ng/mL and 1217 (721–1822) ng/mL, respectively. The corresponding T_max_ values were 2.3 (1.5–4.0) h and 2.3 (1.5–4.0) h, respectively. Elimination half-lives (t_1/2_) were 3.6 (2.7–4.2) h and 3.6 (2.6–4.3) h, respectively. C_max_ for 100 µg LSD was 2.1 (1.1–3.6) ng/mL. T_max_ was 1.4 (0.5–3.5) h. T_1/2_ was 3.5 (2.3.–4.8) h. C_max_ for 20 mg psilocybin was 17 (9.6–34) ng/mL. T_max_ was 2.1 (1.0–5.0) h. T_1/2_ was 2.3 (1.5–2.9) h.

**Table 2:** Pharmacokinetic parameters [geometric mean (95% CI), range] of parent substances and their metabolites.

		C_max_ (ng/mL)	t_max_ (h)	t_1/2_ (h)	AUC_24_ (ng·h/mL)	AUC_∞_ (ng·h/mL)	CL/F (L/h)	V_z_/F (L)
Mescaline 300 **mg (*n*** = **16)**								
	Mescaline	858 (769–992)	2.3 (1.9–2.9)	3.6 (3.5–3.8)	6461 (6028–7022)	6558 (6117–7132)	37 (34–40)	188 (173–209)
		600–1284	1.5–4.0	2.7–4.2	4780–7981	4842–8113	30–50	145–253
	TMPAA	786 (710–905)	2.4 (2.1–3.2)	3.7 (3.4–4.0)	5840 (5274–6676)	6016 (5456–6824)	43 (39–49)	228 (201–270)
		516–1063	1.5–5.1	2.6–4.8	4190–8644	4276–8783	30–61	154–230
	NAM	34 (11–100)	2.6 (2.3–3.2)	2.2 (2.1–2.4)	189 (8–707)	196 (12–712)	1488 (1184–3097)	4754 (3946–9129)
		8.0–357	1.5–4.0	1.7–2.7	44–2782	49–2790	104–5916	369–17115
Mescaline 500 **mg (*n*** = **16)**								
	Mescaline	1217 (1084–1426)	2.3 (1.9–3.0)	3.6 (3.3–3.8)	8974 (8209–10178)	9115 (8344–10315)	45 (39–53)	213 (185–257)
		721–1822	1.5–4.0	2.6–4.3	4238–11470	4400–11570	35–92	143–412
	TMPAA	1120 (986–1336)	2.5 (2.0–3.4)	3.5 (3.2–3.9)	8061 (7149–9592)	8235 (7312–9785)	53 (46–64)	266 (234–320)
		646–1865	1–6	2.3–4.8	4421–12570	4470–12702	34–98	165–445
	NAM	62 (40–175)	3.2 (2.8–3.9)	2.1 (2.0–2.2)	369 (163–1258)	377 (168–1265)	1285 (1080–2956)	3892 (3227–8995)
		18–461	1.5–6.0	1.7–2.4	73–4067	77–4077	119–6304	382–1932
LSD 100 **µg (*n*** = **32)**								
	LSD	2.1 (1.9–2.4)	1.4 (1.3–1.8)	3.5 (3.3–3.8)	14 (13–17)	14 (13–17)	6.5 (6.0–8.2)	33 (30–38)
		1.1–3.6	0.5–3.5	2.3–4.8	6.0–27	6.0–28	3.3–15	19–62
	O-H-LSD	0.15 (0.14–0.16)	4.8 (4.5–5.3)	7.1 (6.8–7.7)	1.8 (1.7–2.0)	2.0 (1.9–2.3)	49 (46–56)	505 (473–568)
		0.87–2.0	3.1–7	5.1–9.7	1.1–2.6	1.3–3.3	31–81	340–802
Psilocybin 20 **mg (*n*** = **32)**								
	Psilocin	17 (15–19)	2.1 (1.9–2.4)	2.3 (2.1–2.4)	84 (76–92)	85 (78–94)	155 (145–177)	505 (467–602)
		9.6–34	1–5	1.5–2.9	46–129	47–130	102–282	259–938
	Psilocin glucuronide	70 (65–81)	4.4 (4.0–5.0)	3.2 (2.1–3.6)	571 (536–633)	608 (570–678)	41 (38–45)	190 (176–223)
		43–127	3–8	2.1–4.8	379–939	408–1007	24–61	99–332
	4-HIAA	86 (81–93)	1.8 (1.6–2.3)	2.1 (2.0–2.4)	327 (310–356)	337 (320–366)	37 (35–40)	116 (103–132)
		50–134	0.5–5	0.7–3.2	177–475	186–483	26–67	38–217

*AUC* area under the plasma concentration-time curve, *AUC∞* AUC from time zero to infinity; AUC24, from time 0-24 h, *CL/F* apparent total clearance, *Cmax* maximum observed plasma concentration; total, after deglucuronidation (unconjugated + glucuronide); unconjugated, *t1/2* plasma half-life, *tmax* time to reach Cmax, *95%CI* 95% confidence interval, *Vz/F* apparent volume of distribution, *4-HIAA* 4-hydroxyindole-3-acetic acid, *O-H-LSD* 2‐oxo‐3‐hydroxy LSD, *TMPAA* 3,4,5-trimethoxyphenylacetic acid, *NAM* N-acetyl mescaline; data are geometric mean with 95% confidence interval of the mean and range.

### Blinding

Attributions of sessions to the four conditions that were guessed by the participants are shown in Supplementary Table S7. Overall, the participants did not unequivocally distinguish mescaline, LSD, and psilocybin during the experience nor after the study. The high mescaline 500 mg dose was correctly identified by 53.3% of the participants during the session and by 81.2% after the study, and was most commonly mistaken for LSD (33.3%) at t = 3 h. The low 300 mg mescaline dose was correctly identified by 50% of the participants during the session and by 68.7% after the study, and was most commonly mistaken for either LSD or placebo (both 18.7%) at t = 3 h. LSD was correctly identified by 58.1% of the participants during the session and by 68.7% after the study, and was most commonly mistaken for either psilocybin or the 300 mg mescaline dose (both 16.1%) at t = 3 h. Psilocybin was correctly identified by 48.4% of the participants during the session and by 78.1% after the study, and was most commonly mistaken for LSD (25.8%) at t = 3 h. Placebo was correctly identified by 96.7% of participants during the session and by 96.8% after the study. One participant mistook placebo for the 300 mg mescaline dose at the end of the study.

## Discussion

The present study directly compared the acute effects of mescaline, LSD, and psilocybin within the same healthy participants. Contemporary research has mostly focused on investigating a single psychedelic substance. Comparisons of serotonergic psychedelics are lacking, except for one recently published study that directly compared LSD and psilocybin [24]. Given the renewed interest in psychedelic substances, systematic comparisons of their acute subjective effects, autonomic effects, and pharmacokinetics are crucial. The present study was the first to compare three classic psychedelics with a randomized, double-blind, placebo-controlled, within-subject design and the first to establish equivalent doses.

We hypothesized that mescaline, LSD, and psilocybin would induce comparable subjective effects due to their shared 5-HT_2A_ receptor agonism. We also hypothesized that mescaline would display more pronounced cardiostimulant properties than LSD and psilocybin because of its activity at adrenergic receptors.

Subjective effects of equivalent doses of the three substances (500 mg mescaline, 100 µg LSD, and 20 mg psilocybin) were similar across various acute effect rating scales. Interestingly, on the AMRS, the condition that caused the highest level of “inactivity” was the low 300 mg mescaline dose. In summary, subjective effects of mescaline, LSD, and psilocybin at equivalent doses were comparable.

The three substances differed in their pharmacokinetics and associated durations of action. As previously reported [24], the acute effects of LSD lasted longer than those of psilocybin in the present study. As expected, effects of 500 mg mescaline lasted longer than those of LSD. However, contrary to our expectation, the longer effect duration of 500 mg mescaline compared with LSD was attributable to its longer time to reach maximal plasma concentrations and subjective effects, whereas the plasma elimination half-life and associated comedown of the subjective effects were similar for mescaline and LSD. Thus, mescaline and LSD had similar plasma elimination half-lives ( ~ 3.5 h) but the t_max_ of the mescaline plasma concentration was approximately 1 h longer than that of LSD. These pharmacokinetic differences between the two substances may be the only clinically relevant pharmacological distinctions between mescaline and LSD. The pharmacokinetics of mescaline were found to be dose-proportional with linear elimination kinetics. Furthermore, there was a close relationship between plasma concentrations of mescaline and its subjective effects within participants, similar to LSD and psilocybin.

The present study was the first to accurately determine the pharmacokinetics of mescaline in humans in a large study using validated analytical methods. The half-life of mescaline was previously reported to be 6 h [42, 43]. However, the true plasma half-life in the present study was only 3.6 h. Notably, the previous estimate was derived from a study that used a small sample and that reported the elimination of ^14^C-labeled radioactive mescaline and any metabolites [42, 43], thereby overestimating the true elimination half-life of mescaline alone.

Autonomic effects of mescaline were comparable to those of LSD and psilocybin [24, 31, 44–48]. However, in the present study, psilocybin induced a significantly higher diastolic blood pressure response than LSD. This finding aligns with greater increases in blood pressure after psilocybin compared with LSD in a previous study [24]. Conversely, LSD showed a trend toward an increase in heart rate compared with psilocybin. Notably, the increase in heart rate in response to the low 300 mg mescaline dose exceeded the increase in heart rate in response to the high 500 mg mescaline dose. When combining elevations of heart rate and blood pressure using the rate pressure product, overall cardiovascular stimulation was comparable for all three substances. No differences were seen in the increases in body temperature or pupil size between substances. Altogether, autonomic effects of mescaline, LSD, and psilocybin were moderate, transient, and not a safety concern. All three substances induced significantly more adverse effects compared with placebo. Mescaline (*n* = 32) was the only substance that induced significant subacute adverse effects (12–24 h) compared with placebo, which may be attributable to its later effect onset and longer duration of action. The number and type of systematically assessed and spontaneously reported adverse effects were comparable to those that were previously reported in a larger pooled analysis of the safety of LSD in healthy participants [46]. In conclusion, the tolerability of mescaline, LSD, and psilocybin was found to be comparable when these substances were used at psychoactive-equivalent doses. The present study reports the following dose equivalence: 500 mg mescaline hydrochloride = 100 µg LSD base = 20 mg psilocybin dihydrate. These results may be helpful for dose finding in future studies and facilitate interpretations of clinical results that are obtained in psychedelic research.

In the present study, blinding across substances was largely sustained during the peak/plateau phase and to a lesser degree even after the study. The condition with the highest probability of being correctly identified was placebo. However, no condition was identified correctly in 100% of the cases, not even placebo at the end-of-study visit. The high 500 mg mescaline dose was never mistaken for psilocybin during the session at t = 3 h and was never mistaken for placebo after the study. LSD and psilocybin were never mistaken for placebo at either t = 3 h or after the study. Placebo was mistaken for 500 mg mescaline by one participant at t = 3 h and was mistaken by another participant for 300 mg mescaline after the study. At t = 3 h, the high mescaline dose was most commonly mistaken for LSD and the low mescaline dose was most commonly mistaken for either LSD or placebo. LSD was most commonly mistaken for either psilocybin or the mescaline 300 mg dose and psilocybin was most commonly mistaken for LSD. This pattern persisted, though at lower numbers, after the study despite the clear differences in effect durations. These findings indicate that any differences in alterations of consciousness that are induced by mescaline, LSD, and psilocybin are dose-dependent rather than substance-dependent and that their distinct pharmacological profiles [19] do not have a relevant influence on the subjective experience. The present study further supports the view that all three substances primarily exert their psychedelic effects through agonistic activity at 5-HT_2A_ receptors [24, 31, 49, 50].

In the present study, both 500 mg mescaline and LSD, but not psilocybin, enhanced circulating oxytocin. Therefore, the present study was the first to document elevated plasma oxytocin levels in response to mescaline as it was previously shown for LSD [24, 27, 28] and psilocybin [24]. In fact, 500 mg mescaline was the strongest releaser of oxytocin among the psychedelics that were tested herein. None of the substances altered plasma BDNF concentrations compared with placebo. It remains unclear whether the use of plasma samples (as opposed to serum samples) is suitable for measuring effects of psychedelics on BDNF concentrations.

The strengths of the present study include its evaluation and use of equivalent doses of three classic psychedelics in a within-subjects design, compared with placebo and in a double-blind laboratory setting. A large study sample was used, with equal numbers of male and female participants. Plasma substance concentrations of all compounds were determined at short intervals up to 24 h. All substances were analyzed with validated analytical methods. As for its limitations; we failed to achieve instant dose equivalence, leading to a subsequent increase in the mescaline dose from 300 to 500 mg. The study thus tested two doses of mescaline against LSD and psilocybin. The comparison of the low and high mescaline doses was between-subjects and their allocation was neither random nor blinded. The study used a highly controlled hospital setting and included only healthy participants, most of whom were experienced psychedelic drug users. Therefore, patients who undergo psychedelic therapy may respond differently to mescaline, LSD, or psilocybin. Lastly, our psychometric instruments may not have been sufficiently sensitive to capture the complex phenomenology of these substances. Subtle qualitative subjective effect differences between mescaline, LSD, and psilocybin may not necessarily be excluded.

## Conclusion

We found no evidence of qualitative differences in altered states of consciousness that were induced by 500 mg mescaline, 100 µg LSD, and 20 mg psilocybin. The substances showed relevant differences in their durations of action. This study supports dose finding for research and psychedelic-assisted therapy.

### Supplementary information

Supplemental Material

CONSORT Flow Chart
