# Supplementary material for: Comparative acute effects of mescaline, lysergic acid diethylamide, and psilocybin in a randomized, double-blind, placebo-controlled cross-over study in healthy participants
Source: Neuropsychopharmacology. 2023 May 25;48(11):1659–67. doi: 10.1038/s41386-023-01607-2 (PMC10517157; doi:10.1038/s41386-023-01607-2)
Supplement: Supplementary file 1 — Supplemental Material [file 41386_2023_1607_MOESM1_ESM.docx]

**Supplement**

**Methods**

*Subjective drug effects measurements*

*Visual Analog Scale (VAS)*

Subjective effects were assessed repeatedly using a visual analog scale (VAS) [1, 2] 1 h before and 0, 0.25, 0.5, 0.75, 1, 1.5, 2, 3, 3.5, 4, 5, 6, 7, 8, 9, 10, 11, 12, 14, 16, and 24 h after drug administration. The VAS consisted of the following items: “any drug effect”, “good drug effect”, “bad drug effect”, “stimulated”, “anxiety”, “nausea”, “visual alterations”, “auditory alterations”, “sounds seem to influence what I see” (= audio-visual synaesthesia), “altered perception of time”, “the boundaries between myself and my surroundings seem to blur” (= ego-dissolution), “I am gaining insight into connections that previously puzzled me” (= insight). These unidirectional items were presented as 100-mm horizontal lines (0–100%) labelled “not at all” on the left and “extremely” on the right [1, 3]. Additionally, the scale contained three bidirectional items, i.e. “talkative”, “open”, and “trust”, labelled “normal” in the middle at 50 mm, “not at all” on the left (0 mm), and “extremely” (100 mm) on the right. The primary VAS outcome measures were “any drug effect”, “good drug effect”, and “bad drug effect”. These VAS items have been repeatedly applied in the previous years and have proven to be sensitive to psychedelic substances, especially in a low to moderate dosing range [1, 2, 4, 5]. The items were further used for the pharmacokinetic-pharmacodynamic modeling as similarly done previously [4, 5]. The VAS is a low-effort measure which can be completed rapidly by the participant during a psychedelic experience. Despite its simplicity it possesses high sensitivity and allows mapping of the subjective effects over time. Due to the inherent distractibility of the psychedelic state, more complex assessments must be performed at the end of the session. The VAS item “any drug effect” is a measure to characterize the general effect intensity and time course. The VAS item “good drug effect” measures effects which are subjectively considered positive and is interrelated with other items such as “stimulated”. The VAS item “bad drug effect” is a measure for any negative effects and is related to “anxiety”. Typically, “bad drug effects” tend to occur at higher doses or plasma concentrations according to previous PK-PD analyses [4, 5]. The VAS items “sounds seem to influence what I see”, “the boundaries between myself and my surroundings seem to blur”, and “I am gaining insight into connections that previously puzzled me” were presented in full sentences obtained from the 5D-ASC (item no. 20, no. 71, and no. 69). This approach has been used previously [1, 4, 6-8] to create comprehensible definitions of complex terms like “synaesthesia”, “ego-dissolution”, or “insight”.

The VAS was administered each time plasma blood concentrations were measured to allow for pharmacokinetic-pharmacodynamic modeling.

*5 Dimensions of Altered States of Consciousness (5D-ASC) scale*

The 5 Dimensions of Altered States of Consciousness (5D-ASC) scale [9, 10] was used as the primary outcome measure and was administered 24 h after drug administration to retrospectively rate the psychedelic experience. The 5D-ASC contains 94 items presented on visual analog scales which may be clustered to five subscales/dimensions [9] and 11 lower-order subscales [10]. The 5D-ASC dimension “oceanic boundlessness” (27 items) measures derealization and depersonalization associated with positive emotional states, ranging from heightened mood to euphoric exaltation. The corresponding lower-order subscales include “experience of unity,” “spiritual experience,” “blissful state,” “insightfulness,” and “disembodiment”. The dimension “anxious ego dissolution” (21 items) summarizes ego-disintegration and loss of self-control phenomena associated with anxiety. The corresponding lower-order scales include “impaired control of cognition” and “anxiety.” The dimension “visionary restructuralization” (18 items) consists of the lower-order scales “complex imagery,” “elementary imagery,” “audio-visual synesthesia,” and “changed meaning of percepts”. Two additional dimensions describe “auditory alterations” (15 items) and “reduction of vigilance” (12 items). The total 3D-ASC score is calculated by adding the scores of the three main dimensions “oceanic boundlessness”, “anxious ego-dissolution”, and “visionary restructuralization” and can be interpreted as a measure for the general intensity of alteration of the mind [7]. The scale is well-validated in German [9] and many other languages and widely used to characterize the subjective effects of various psychedelic substances. In particular, the scale has been used by most research groups investigating psychedelics [1, 2, 11-15]. Furthermore, acute ratings on the 5D-ASC after administration of psilocybin could predict long-term effects of psychedelic treatments in patients [16, 17]. Ratings on the 5D-ASC have been shown to closely correlate with ratings on the Mystical Effects Questionnaire (MEQ, see below) [7] which is primarily used by research groups in the US [17].

*Mystical Effects Questionnaire (MEQ30)*

Mystical experiences were assessed 24 h after drug administration using the 100-item States of Consciousness Questionnaire (SOCQ) [7, 18] which includes the 43-item Mystical Effects Questionnaire (MEQ43) [18], 30-item Mystical Effects Questionnaire (MEQ30) [19], and subscales for “aesthetic experience” and negative “nadir” effects. The MEQ has been used in numerous clinical trials with psilocybin [17, 18, 20-26]. The MEQ items provide scale scores for seven domains of mystical experiences: Internal unity, external unity, sacredness, noetic quality (as real as or more real than everyday reality), deeply felt positive mood, transcendence of time and space, and ineffability/paradoxicality (difficulty describing the experience in words). The sum of all scale scores was used as an overall measure of the mystical-type experience. We also derived the four scale scores of the revised 30-item MEQ: Mystical experience, positive mood, transcendence of time and space, and ineffability [19]. A complete mystical experience was defined as scores ≥ 60% on all MEQ30 factors [19]. For the scale validation see [19]. For an analysis of the interrelation of the MEQ and the 5D-ASC in regard to responses to LSD see [7]. For the German translation of the MEQ30 see online supplement of [7].

*Adjective Mood Rating Scale (AMRS)*

The Adjective Mood Rating Scale (AMRS) [27] was used 1 h before and 3, 6, 9, 12, and 24 h after drug administration. The AMRS is a validated 60-item Likert mood rating scale mainly used in Europe and consists of 15 subscales which in turn can be clustered into 6 higher-level subscales including “activity”, “inactivity”, “extraversion/introversion”, “irritability”, and “anxiety”. The AMRS is suitable for repeated measurements of mood states. In this study, the short German EWL60S version was used [27]. Completing a 60-item scale under the effects of psychedelic substances on paper has proven to be possible but rather difficult due to volatile cognitive effects. The AMRS was included as a secondary supportive measure as its validation as a mood measuring tool is better compared to the VAS. Also, the AMRS yields more defined ratings than the VAS and may corroborate the VAS results (AMRS “well-being” is considered similar to VAS “good drug effect”; AMRS “anxiety” is considered similar to VAS “anxiety”).

**Sample size determination:** Power analysis was performed using PASS®, Hintze J. Kaysville, Utah, US. Using a cross-over design and CV% of 25 based on similar studies in our laboratory [28, 29], a sample size of 23 subjects would achieve 80% power to demonstrate equivalence of effects at a 5% significance level when the true ratio of the means is 1 and the equivalence interval is 0.8–1.25.

**Changes to trial** **plan after trial start:** Outcomes were not changed. After completion of 16 subjects, an interim comparison of the peak subjective “any drug effect” was performed as a measure of the overall subjective effect intensity. Based on lower responses in the mescaline group compared with psilocybin and LSD, the mescaline dose was increased from 300 mg to 500 mg in the subsequent 16 subjects, resulting in the use of two doses of mescaline in a non-random order in a total sample of 32 participants. Thus, the total study sample was increased from the initially planned sample of 24 to 32.

**Randomization:** A computer-generated block randomization was used by the study medication provider to assign the treatments in balanced and random order. The medication provider was not part of the study team. Allocation to the low and high mescaline dose was ordered (300 mg in participants 1–16, 500 mg in participants 17–32).

**Results**

**
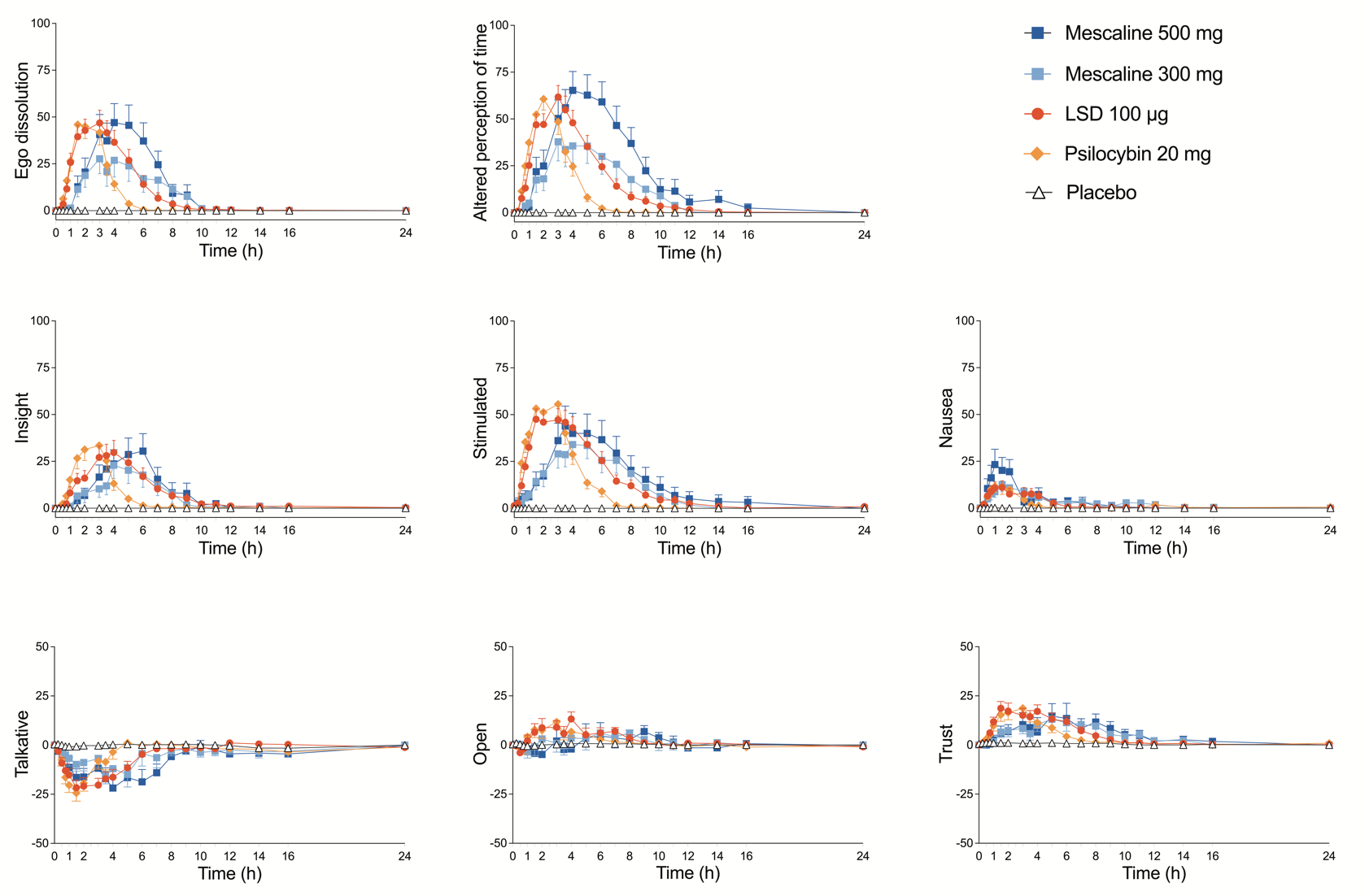
**

**Figure S1.** Acute subjective effects induced by 300 and 500 mg mescaline, 100 µg LSD, 20 mg psilocybin, and placebo over time on the Visual Analog Scale (VAS). The high 500 mg mescaline dose, LSD, and psilocybin induced similar subjective peak effects on all items. The low 300 mg mescaline dose overall induced lower peak effects than the high mescaline dose, LSD, and psilocybin. Substances were administered at t = 0 h. Data are expressed as the mean ± SEM ratings in 32 subjects for LSD and psilocybin, and in 16 subjects for each mescaline dose. The corresponding statistics are presented in Supplementary Table S1.

**

**

**Figure S2.** Acute subjective effects on the Adjective Mood Rating Scale (AMRS) over time. Mescaline, LSD, and psilocybin had a comparable effect on mood. The low 300 mg mescaline dose induced higher ‘inactivity’ scores than psilocybin. Substances were administered at t = 0. Data are expressed as the mean ± SEM change from baseline. The corresponding statistics are presented in Supplementary Table S4.

**

**

**Figure S3.** Effects on pupillary function. All substances similarly increased pupil size **(a**–**b)** relative to placebo. Psilocybin, but not mescaline and LSD, significantly reduced the constriction reaction to a light stimulus compared with placebo **(c)**. Data are expressed as the mean ± SEM in 32 subjects for LSD and psilocybin, and in 16 subjects for each mescaline dose. The corresponding statistics are presented in Supplementary Table S5.

**

**

**Figure S4.** Effects on plasma levels of oxytocin and brain-derived neurotrophic factor (BDNF). Mescaline and LSD significantly increased plasma oxytocin levels compared with placebo. Oxytocin levels were significantly higher after mescaline (n = 32) compared with psilocybin. None of the substances altered plasma BDNF.

**
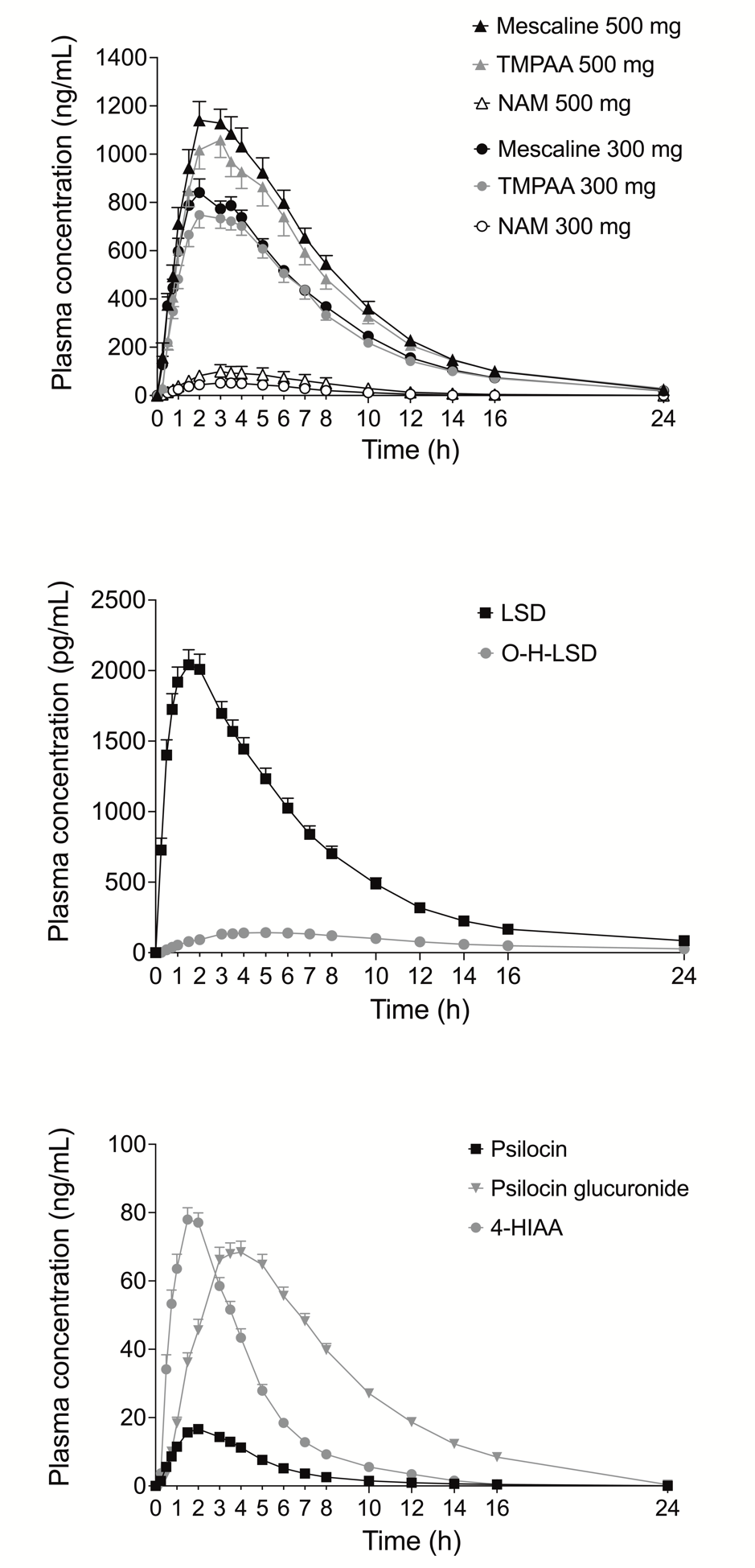
**

**Figure S5.** Plasma concentrations of mescaline, LSD, and psilocin and their metabolites. The corresponding data is presented in Table 2.

|  |  |  |  |  |  |
| --- | --- | --- | --- | --- | --- |

**References**

1 Holze F, Vizeli P, Muller F, Ley L, Duerig R, Varghese N, et al. Distinct acute effects of LSD, MDMA, and D-amphetamine in healthy subjects. Neuropsychopharmacology. 2020;45(3):462-71.

2 Schmid Y, Enzler F, Gasser P, Grouzmann E, Preller KH, Vollenweider FX, et al. Acute effects of lysergic acid diethylamide in healthy subjects. Biol Psychiatry. 2015;78(8):544-53.

3 Hysek CM, Schmid Y, Simmler LD, Domes G, Heinrichs M, Eisenegger C, et al. MDMA enhances emotional empathy and prosocial behavior. Soc Cog Affect Neurosci. 2014;9:1645-52.

4 Holze F, Duthaler U, Vizeli P, Muller F, Borgwardt S, Liechti ME. Pharmacokinetics and subjective effects of a novel oral LSD formulation in healthy subjects. Br J Clin Pharmacol. 2019;85:1474-83.

5 Dolder PC, Schmid Y, Steuer AE, Kraemer T, Rentsch KM, Hammann F, et al. Pharmacokinetics and pharmacodynamics of lysergic acid diethylamide in healthy subjects. Clin Pharmacokinetics. 2017;56:1219-30.

6 Tagliazucchi E, Roseman L, Kaelen M, Orban C, Muthukumaraswamy SD, Murphy K, et al. Increased global functional connectivity correlates with LSD-induced ego dissolution. Curr Biol. 2016;26(8):1043-50.

7 Liechti ME, Dolder PC, Schmid Y. Alterations in conciousness and mystical-type experiences after acute LSD in humans. Psychopharmacology. 2017;234:1499-510.

8 Holze F, Ley L, Muller F, Becker AM, Straumann I, Vizeli P, et al. Direct comparison of the acute effects of lysergic acid diethylamide and psilocybin in a double-blind placebo-controlled study in healthy subjects. Neuropsychopharmacology. 2022;47:1180-87.

9 Dittrich A. The standardized psychometric assessment of altered states of consciousness (ASCs) in humans. Pharmacopsychiatry. 1998;31 (Suppl 2):80-4.

10 Studerus E, Gamma A, Vollenweider FX. Psychometric evaluation of the altered states of consciousness rating scale (OAV). PLoS One. 2010;5:e12412.

11 Carhart-Harris RL, Kaelen M, Bolstridge M, Williams TM, Williams LT, Underwood R, et al. The paradoxical psychological effects of lysergic acid diethylamide (LSD). Psychol Med. 2016;46:1379-90.

12 Dolder PC, Schmid Y, Mueller F, Borgwardt S, Liechti ME. LSD acutely impairs fear recognition and enhances emotional empathy and sociality. Neuropsychopharmacology. 2016;41:2638-46.

13 Bershad AK, Schepers ST, Bremmer MP, Lee R, de Wit H. Acute subjective and behavioral effects of microdoses of lysergic acid diethylamide in healthy human volunteers. Biol Psychiatry. 2019;86(10):792-800.

14 Preller KH, Herdener M, Pokorny T, Planzer A, Kraehenmann R, Stämpfli P, et al. The fabric of meaning and subjective effects in LSD-induced states depend on serotonin 2A receptor activation. Curr Biol. 2017;27:451-57.

15 de Deus Pontual AA, da Motta LG, de Oliveira IR, Palhano-Fontes F, Onias H, Ribeiro S, et al. Systematic review of psychometric instruments used in research with psychedelics. J Psychoactive Drugs. 2022;54:1-10.

16 Roseman L, Nutt DJ, Carhart-Harris RL. Quality of acute psychedelic experience predicts therapeutic efficacy of psilocybin for treatment-resistant depression. Front Pharmacol. 2017;8:974.

17 Griffiths RR, Johnson MW, Carducci MA, Umbricht A, Richards WA, Richards BD, et al. Psilocybin produces substantial and sustained decreases in depression and anxiety in patients with life-threatening cancer: a randomized double-blind trial. J Psychopharmacol. 2016;30(12):1181-97.

18 Griffiths RR, Richards WA, McCann U, Jesse R. Psilocybin can occasion mystical-type experiences having substantial and sustained personal meaning and spiritual significance. Psychopharmacology. 2006;187(3):268-83; discussion 84-92.

19 Barrett FS, Johnson MW, Griffiths RR. Validation of the revised Mystical Experience Questionnaire in experimental sessions with psilocybin. J Psychopharmacol. 2015;29(11):1182-90.

20 MacLean KA, Johnson MW, Griffiths RR. Mystical experiences occasioned by the hallucinogen psilocybin lead to increases in the personality domain of openness. J Psychopharmacol. 2011;25(11):1453-61.

21 Griffiths RR, Johnson MW, Richards WA, Richards BD, McCann U, Jesse R. Psilocybin occasioned mystical-type experiences: immediate and persisting dose-related effects. Psychopharmacology. 2011;218(4):649-65.

22 Griffiths R, Richards W, Johnson M, McCann U, Jesse R. Mystical-type experiences occasioned by psilocybin mediate the attribution of personal meaning and spiritual significance 14 months later. J Psychopharmacol. 2008;22(6):621-32.

23 Garcia-Romeu A, Griffiths RR, Johnson MW. Psilocybin-occasioned mystical experiences in the treatment of tobacco addiction. Curr Drug Abuse Rev. 2015;7(3):157-64.

24 Garcia-Romeu A, Davis AK, Erowid F, Erowid E, Griffiths RR, Johnson MW. Cessation and reduction in alcohol consumption and misuse after psychedelic use. J Psychopharmacol. 2019:269881119845793.

25 Griffiths RR, Johnson MW, Richards WA, Richards BD, Jesse R, MacLean KA, et al. Psilocybin-occasioned mystical-type experience in combination with meditation and other spiritual practices produces enduring positive changes in psychological functioning and in trait measures of prosocial attitudes and behaviors. J Psychopharmacol. 2018;32:49-69.

26 Ross S, Bossis A, Guss J, Agin-Liebes G, Malone T, Cohen B, et al. Rapid and sustained symptom reduction following psilocybin treatment for anxiety and depression in patients with life-threatening cancer: a randomized controlled trial. J Psychopharmacol. 2016;30(12):1165-80.

27 Janke W, Debus G. Die Eigenschaftswörterliste. Göttingen.: Hogrefe; 1978.

28 Hysek, C.M., Fink, A.E., Simmler, L.D., Donzelli, M., Grouzmann, E., Liechti, M.E. Alpha1-adrenergic receptors contribute to the acute effects of 3, 4- methylenedioxymethamphetamine in humans. J Clin Psychopharmacol. 2013;33:658-666.

29 Hysek, C.M., Brugger, R., Simmler, L.D., Brugisser, M., Donzelli, M., Grouzmann, E. et al. Effects of the alpha2-adrenergic agonist clonidine on the pharmacodynamics and pharmacokinetics of 3,4-methylenedioxymethamphetamine in healthy volunteers. J Pharmacol. Exp Ther, 2012;340:286-294.
