# Supplementary figures and images for: Comparative acute effects of mescaline, lysergic acid diethylamide, and psilocybin in a randomized, double-blind, placebo-controlled cross-over study in healthy participants
Source: Neuropsychopharmacology. 2023 May 25;48(11):1659–67. doi: 10.1038/s41386-023-01607-2 (PMC10517157; doi:10.1038/s41386-023-01607-2)

## STUDY

Mescaline: 300, 500 mg  
LSD: 100 µg  
Psilocybin: 20 mg  
Placebo

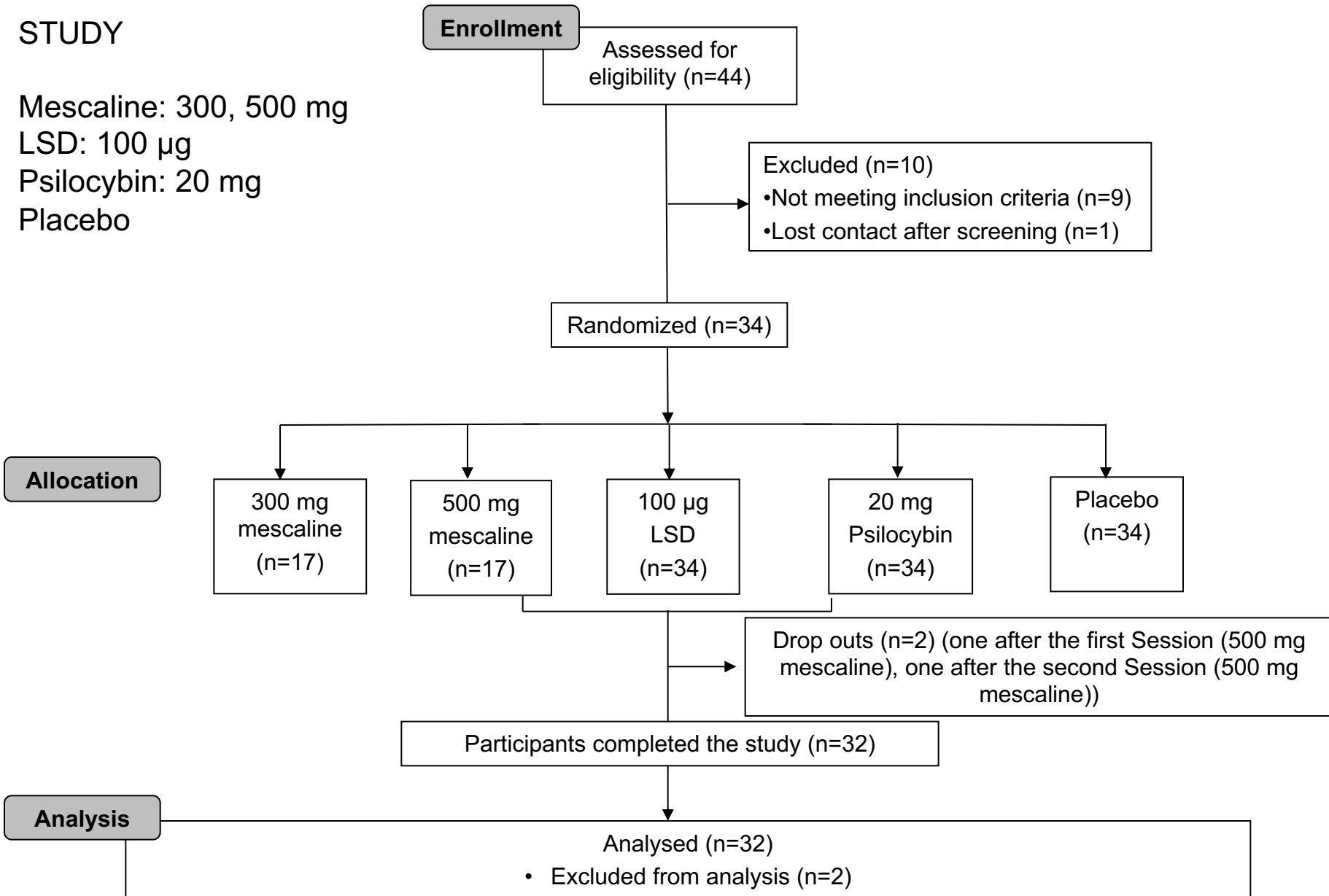

Supplement: Supplementary file 2 — CONSORT Flow Chart [file 41386_2023_1607_MOESM2_ESM.pdf]
